# A New Method to Determine Antioxidant Activities of Biofilms Using a pH Indicator (Resazurin) Model System

**DOI:** 10.3390/molecules28052092

**Published:** 2023-02-23

**Authors:** Young-Teck Kim, Robert Kimmel, Xiyu Wang

**Affiliations:** 1 Department of Sustainable Biomaterials, College of Natural Resources and Environment, Virginia Tech, Blacksburg, VA 24061, USA; 2 Food, Nutrition, and Packaging Sciences Department, College of Agriculture, Forestry and Life Sciences, Clemson University, Clemson, SC 29634, USA

**Keywords:** gelatin, biopolymer film, BHA, ascorbic acid, phytic acid, resazurin

## Abstract

Biopolymeric films were prepared with gelatin, plasticizer, and three different types of antioxidants (ascorbic acid, phytic acid, and BHA) corresponding to different mechanisms in activity. The antioxidant activity of films was monitored for 14 storage days upon color changes using a pH indicator (resazurin). The instant antioxidant activity of films was measured by a DPPH free radical test. The system using resazurin was composed of an agar, an emulsifier, and soybean oil to simulate a highly oxidative oil-based food system (AES-R). Gelatin-based films (GBF) containing phytic acid showed higher tensile strength and energy to break than all other samples due to the increased intermolecular interactions between phytic acid and gelatin molecules. The oxygen barrier properties of GBF films containing ascorbic acid and phytic acid increased due to the increased polarity, while GBF films containing BHA showed increased oxygen permeability compared to the control. According to “a-value” (redness) of the AES-R system tested with films, films incorporating BHA showed the most retardation of lipid oxidation in the system. This retardation corresponds to 59.8% antioxidation activity at 14 days, compared with the control. Phytic acid-based films did not show antioxidant activity, whereas ascorbic acid-based GBFs accelerated the oxidation process due to its prooxidant activity. The comparison between the DPPH free radical test and the control showed that the ascorbic acid and BHA-based GBFs showed highly effective free radical scavenging behavior (71.7% and 41.7%, respectively). This novel method using a pH indicator system can potentially determine the antioxidation activity of biopolymer films and film-based samples in a food system.

## 1. Introduction

For the last 20 years, many scientists have been attracted to protein-based biopolymeric films and coating technology due to their thermo-reversible, non-toxic, biodegradable properties, and extensive applications. Gelatin is an inexpensive and widely used substance prepared through collagen’s thermal denaturation that is isolated from skin and bones using a very dilute acid or alkali [1,2,3,4]. Gelatin is a heterogeneous mixture of single or multi-stranded polypeptides [4,5]. Thus, solutions undergo coil–helix transition followed by aggregation of the helices by the formation of a collagen-like right-handed triple-helical structure, resulting in a well-ordered matrix [5,6,7,8]. Based on “free volume theory”, which is about the diffusion of small biomaterials in a polymer matrix, gelatin has been used as an excellent film-forming material as well as a good carrier material for holding low molecular weight active agents such as antioxidants or drugs [1,9,10,11,12].

The development of active biopolymer films has always been related to the reduction of moisture, selective gas transfer, oxidation, or the respiration in food systems, as well as migration of active agents in order to prolong the shelf-life of food products [13,14,15,16]. It is well-known that biopolymeric films have low oxygen permeability and high water vapor permeability (WVP) properties due to the abundant hydroxyl groups and free volume made during film formation. Permeability studies have been most extensively performed due to the importance of water and oxygen in deteriorative reactions and the ease of measurement [14,15,16]. The incorporation of antioxidants, which may be organic lipid- or water-soluble substances of either synthetic or natural origin that can prevent or delay the progress of lipid oxidation, has increased the potential application of biopolymeric films [17,18,19,20]. The use of commercial antioxidants such as ascorbic acid, phytic acid, tocopherol, butylated hydroxytoluene (BHT), and butylated hydroxyanisole (BHA) has already become a common trend in active packaging film due to economic issues and safety [5,21,22].

**Figure 1:**
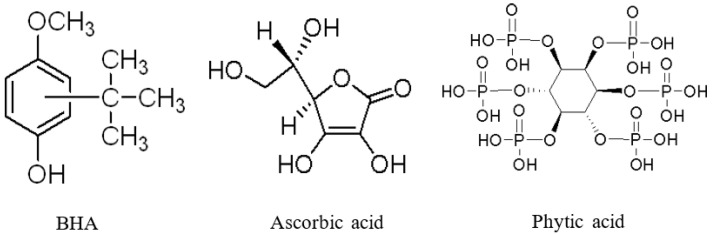
Chemical structures of antioxidants used in our study.

In this study, to fabricate antioxidative biopolymer films that have different antioxidation mechanisms, three typical water-soluble antioxidants were used (Figure 1). Phytic acid (PA) is a natural and powerful antioxidant that can isolate metallic ions [23,24]. Phytic acid chelate ions inhibit the formation of hydroxyl radicals, thus making them catalytically inactive. BHA is well known as a common antioxidant, while ascorbic acid plays either the role of antioxidant or prooxidant depending on the concentration [25,26,27,28]. The antioxidant activities of ascorbic acid are mainly based on the radical scavenging ability intrinsic to its resonance structure.

Traditional methods for determining the antioxidant activity of films are as follows: TBARS test (most common and reliable), peroxide value, enzymatic evaluation, physical changes (color and odor), and radical scavenging test [29,30,31,32,33,34,35]. These methods are very time-consuming and require highly trained skills; as a result, they have not been thoroughly employed for the food system using biopolymer film due to the complexity of food and technical difficulties. Thus, a simple method to determine antioxidation activity of film has been demanded to ensure food safety.

**Figure 2:**
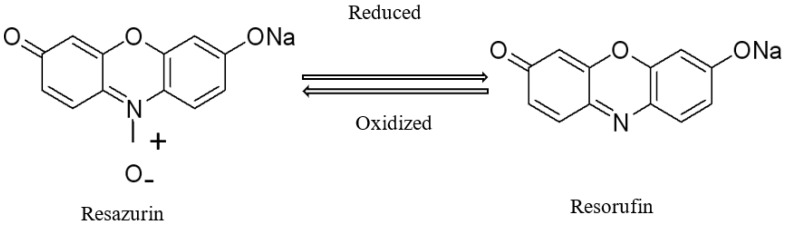
The chemical reaction structures of resazurin and resorufin as a pH indicator. The oxidized form corresponds to resazurin and the reduced form to resorufin. Its change is measured at 517 nm.

The alamarBlue dye, also known as resazurin, is a colorimetric pH indicator based on redox potential (Figure 2). The system incorporates an oxidation–reduction (REDOX) indicator that can both fluoresce and change color in response to pH changes resulting from biological lipid oxidation [36]. Owing to this typical property, it has been spread as a biological microorganism growth assay. For example, the test has been accepted for use in determining the quality of meat [37]. It was reported that the resazurin reduction time could be used as a rapid method to estimate bacterial spoilage load in beef stored at 4 °C [34]. Moreover, the assay has been widely used in medical sciences to determine the minimum inhibitory concentration (MIC) values of various anti-microbial compounds [37]. On the other hand, resazurin has never been used as an indicator expressing antioxidant activity, such as the degree of lipid oxidation for the biopolymeric film. The properties of resazurin may help determine the process of lipid oxidation of a food system as a simple colorimetric indicator. In our study, the colorimetric alamarBlue (resazurin) was added into the model food system, which was prepared with mainly agar, soybean oil, and emulsifier. The last two components could be used for lipid sources. This colorimetric method is a relatively simple, rapid, low-cost, and appropriate technology that is not time-consuming and does not require expensive instrumentation compared with traditional labor-intensive methods.

Therefore, the ultimate objective of this paper is to develop a simple and novel method for determining antioxidant activity of film using Redox potential, while discussing the obstacles that need to be overcome in order to make an extensive application of this model food system.

## 2. Results and Discussion

### 2.1. Mechanical Properties

As shown in Table 1, adding phytic acid increased at most by 35% and 40% in tensile strength and break energy, respectively, compared with the control. This result is attributed to the increased intermolecular bonds induced by the functional groups of phytic acid and gelatin molecules. Interestingly, BHA-based GBF showed lower elongational properties than all others due to the increased brittleness induced by the phenolic chemical structure of BHA, while other GBFs showed no significant change compared with the control. Adding ascorbic acid and BHA into GBFs did not show a plasticizing effect and modification of toughness that is expressed by the total area (energy to break) in the stress–strain curve.

The literature shows that [38] higher flexibility and lower brittleness are highly desirable for successful flexible packaging applications. As a matter of fact, the mechanical properties of biopolymer films depend on material characteristics such as their structural matrix [39,40]. In the case of gelatin, mechanical properties correspond to the bloom index (similar to viscosity) and plasticizer effect. Furthermore, the bloom index results from the structure of the polymer and, particularly, its molecular strength, geometry, molecular weight distribution, and the type of position of its lateral groups [41]. Mechanical properties are also linked to the film-forming conditions. Values were mainly in agreement for the deformation at the break of gelatin film, which was dependent on the bloom index because of the greater extensibility of films containing higher amounts of triple-helix structures. In contrast, other treatments, such as enzyme process and chemical reaction, did not show this effect on mechanical properties [42]. The incorporation of ferulic acid as an antioxidant into the soybean isolate film increases the TS value and percent elongation due to the cross-linking effect of ferulic acid [37]. The values of mechanical properties of films produced in our work showed a similar pattern to previous results [37,43,44].

### 2.2. Permeability

Water vapor permeability (WVP) is the function of permeance, environmental condition, thickness, free volume of matrix, and hygroscopic characteristics of a film [45,46]. Among all GBFs, the incorporation of 0.6% phytic acid showed the lowest WVP (highest water barrier property), while ascorbic acid-based GBFs showed a simple, gradual increase as a function of concentration (see Table 2). The possible mechanism for this phenomenon is the electrostatic cross-linking between six phosphate functional groups in phytic acid and amino groups in the gelatin structure. These WVP results were well in agreement with the mechanical properties, oxygen permeability, and WVP of GBFs. In natural products (e.g., cereals or soybeans), phytic acid has been found in the electrostatic complex with protein, which makes the protein less digestible [47]. A similar phenomenon was observed in previous literature, as well [37,48].

**Figure 3:**
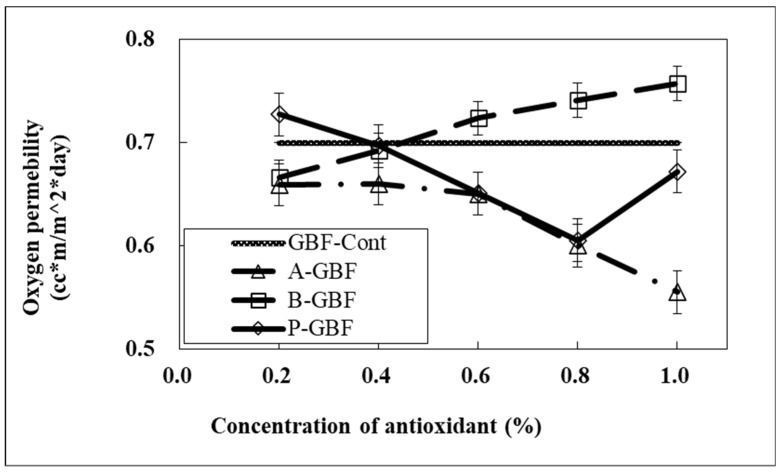
Oxygen permeability of GBFs incorporating antioxidants at various concentrations (50% RH, 23 °C). Note that GBF-Cont represents pure GBF while A-, B-, and P- stand for ascorbic acid, BHA, and phytic acid, respectively. Bars indicate the standard deviation.

It was of interest that the oxygen permeability (OP) measured by Oxtran (Mocon, Co., Brooklyn Park, MN, USA) was decreased when phytic acid and ascorbic acid were incorporated into GBFs, while BHA-based GBFs showed the opposite result (Figure 3). The effect of phytic acid on OP agrees with mechanical properties and WVP induced by the cross-linking effect on chemical structures. In addition, biopolymer films have relatively low OP due to the abundance of hydroxyl groups. Phytic acid has six phosphate functional groups, which caused the increase of polarity in GBFs in correspondence to the improved oxygen barrier property.

The structural effect of ascorbic acid as an oxygen scavenger has been well-studied in much of the literature [24,25]. Ascorbic acid consists of an enediol structure conjugated with a carbonyl group in a lactone ring [49]. In the presence of oxygen, ascorbic acid is degraded primarily to dehydroascorbic acid via its mono-anion, resulting in lowering oxygen permeability in gelatin film due to the high polarity of the chemical composition of ascorbic acid.

Meanwhile, BHA has not been known as an oxygen scavenger but rather as a free radical and hydroxyl peroxide scavenger in bio-systems [27]. Therefore, BHA-based GBFs could not increase the oxygen barrier property compared to the control film.

### 2.3. Calibration of AES-R Systems

The color changes of AES-R were recorded for 14 days to investigate the effect of 3 different model systems: (1) only agar (A-R), (2) a combination of agar and emulsifier (AE-R), and (3) a combination of agar, emulsifier, and soybean oil (AES-R), on lipid oxidation measurement through comparison to the control system (AES-R covered with the control film, AES-R-Cont). Resazurin was added to each system as a colorimetric pH indicator.

Recording color changes to determine the lipid oxidation process has been used for meat and poultry sciences and irradiation studies for a long time because the oxidation of pigments can catalyze the oxidation of lipids [50]. Several studies reported that free radicals generated by irradiation could also react with myoglobin or hemoglobin, resulting in changes in the color of irradiated samples [51,52]. However, these technologies have been used only for recording the direct color change of a target sample induced by treatments.

Meanwhile, the color change of pH indicators showing the reduction and oxidation reaction potential has been used as a microbial growth indicator in the biology and medical fields based on the detection of metabolic activity of cells [34,36,37,53]. This redox potential pH indicator (resazurin) both fluoresces and changes color, transitioning from blue to red, in response to chemical reduction induced by metabolic activity [53]. Lipid oxidation produces various acid molecules as secondary and tertiary byproducts while breaking down, implying that the lipid oxidation may corresponding to the Redox potential.

**Figure 4:**
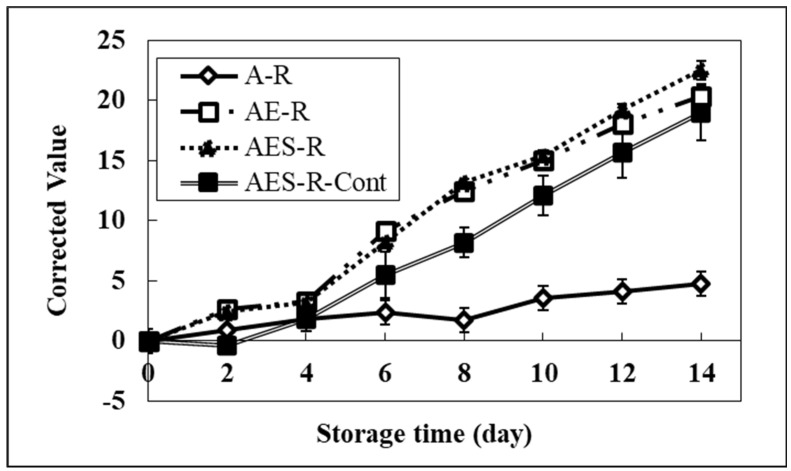
Brightness (“L”) of model systems containing resazurin for 14 days. Note that it was used for calibrating our system. A-, AE-, and AES- stand for agar, agar and emulsifier, and agar, emulsifier, and soybean oil, respectively. AES-R-Cont represents AES-R system covered with pure GBF. Bars indicate the standard deviation.

As shown in Figure 4, the brightness of model systems incorporating resazurin was severely affected by the addition of emulsifier and soybean oil into the model system compared to the A-R system. After 6 days, a significant change in the brightness of all systems started compared to the A-R system. The brightness values of all systems at 14 days were 20.3 (AE-R), 22.5 (AES-R), 19.0 (AES-R-Cont), and 4.7 (A-R). These results suggested that the brightness of a model food system containing a lipid portion might correspond to the degree of the lipid oxidation progress. Therefore, the A-R system, having no lipid portion, did not show a significant change in brightness. The value of the A-R system at day 14 might reflect the stability of resazurin, showing the baseline of our system. Furthermore, the AES-R-Cont showed a lower value than the other two model systems (AE-R and AES-R), implying pure GBF plays the role of an oxygen barrier layer, inducing a lower possibility of lipid oxidation. These results strongly suggest that this system can help determine the progress of lipid oxidation in a food packaging system.

**Figure 5:**
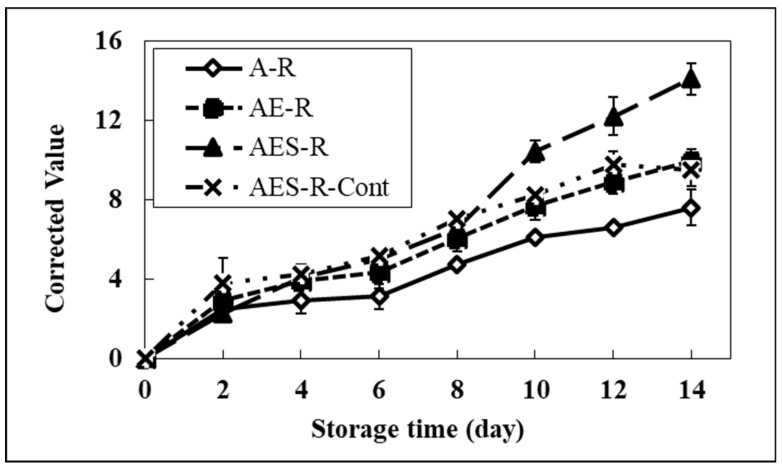
Redness (“a”) of model systems containing resazurin for 14 days. Note that it was used for calibrating our system. A-, AE-, and AES- stand for agar, agar and emulsifier, and agar, emulsifier, and soybean oil, respectively. AES-R-Cont represents AES-R system covered with pure GBF. Bars indicate the standard deviation.

As shown in Figure 5, in the calibration system, a significant increase of redness in all systems compared to the A-R system was observed from the 4th day. On day 14, the redness of the AES-R system containing the highest lipid portion (oil and emulsifier) was 14.1. This redness was 85% and 49% higher than those of the A-R system and AES-R-Cont, respectively. It was interesting that the AE-R system also showed changes in brightness and redness compared to the A-R system. This phenomenon can be explained by the fact that the emulsifier (Tween 80) used in our study is composed of two portions in the structure; one is fatty acid (oleic acid) and the other one is hydrophilic portion (polyoxylated sorbitan) as nonionic surfactant characteristics. Therefore, the change of color in the AE-R system corresponds to the degradation of the lipid portion (oleic acid), implying that the emulsifier itself could be a good source of lipid in the model system. These results suggested that the redness of each system was strongly affected by lipid oxidation, indicating that the REDOX potential using a pH indicator can be useful for measuring the degree of lipid oxidation in food systems.

Even though the redness and brightness showed similar patterns, the color change rate is significantly different. In general, resazurin is a substrate that shows color changes in response to pH changes, from neutral (blue) to acid (red). Brightness could be affected by not only resazurin but also oil color and the oil’s byproduct colors. A simple test with the direct addition of BHA and ascorbic acid into the AES-R system was conducted to confirm these color changes. The AES-R system containing BHA did not affect the brightness due to its intense antioxidation activity, whereas the high concentration of ascorbic acid in the AES-R system dramatically increased the brightness due to its strong prooxidant activity. Thus, we decided to record the redness for further study to determine the lipid oxidation process. Likewise, this redness is in strong agreement with DPPH radical scavenging results.

### 2.4. Antioxidation Activity of GBFs Using the AES-R System

**Figure 6:**
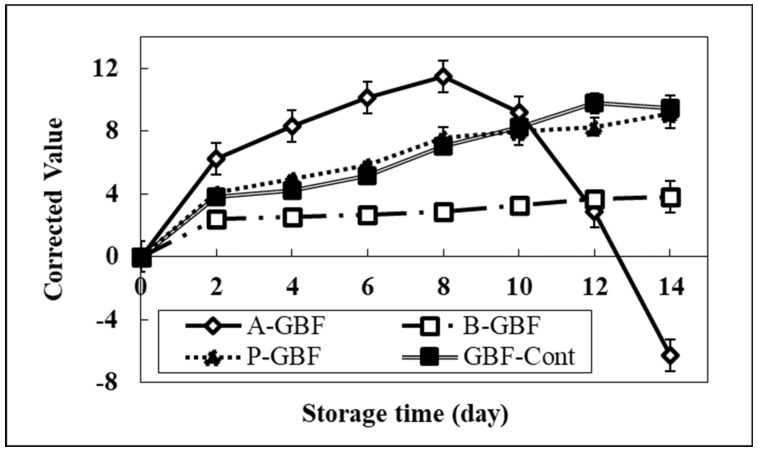
Redness of AES-R systems treated with various GBFs having 0.6% of BHA and phytic acid and 1% of ascorbic acid for 14 days. Herein, all other redness information of GBFs is in Supplemental Figures. Note that GBF-Cont s represents pure GBF while A-, B-, and P- stand for ascorbic acid, BHA, and phytic acid, respectively. Bars indicate the standard deviation.

The redness of the AES-R system covered by antioxidants-based GBFs was recorded for 14 days and then compared with the AES-R-Cont system. Antioxidants were used at three different concentrations (0.2, 0.6, and 1.0% of gelatin). In general, the redness (a-value) of all tested AES-R systems increased as a function of storage time due to the lipid oxidation process, resulting in a decrease in pH. Figure 6 shows the typical curve of the lipid oxidation process in the AES-R system tested at 0.6% phytic acid and BHA, and 1.0% ascorbic acid concentration. Compared to the control, the AES-R system treated with phytic acid-based GBFs did not show any effect on a-value regardless of phytic acid concentration, even though phytic acid is a strong natural antioxidant agent.

Compared with the control, GBFs incorporating 0.6% ascorbic acid showed no significant change in redness up to Day 8 (see Supplemental Figures), then its redness dropped dramatically after 8 days passed. As shown in Figure 6, the GBF containing 1% ascorbic acid showed the highest redness among all others up to Day 8, then dropped dramatically after Day 8. This implies that the higher ascorbic acid concentration played the prooxidant role, resulting in the fast lipid oxidation process at the beginning of storage time. In addition, the more rapid drop of acidity (redness) may correspond to the instability of resazurin induced by the high quantity of ascorbic acid.

As a function of BHA concentration, the GBF with a higher level of BHA showed a very slow color change from blue to red compared to the AES-R-Cont. This phenomenon could be explained by different antioxidation mechanisms. There are several factors that accelerate lipid oxidation. Considering free radicals and metal ions as oxidation factors, the free radical activity can be inhibited by BHA, while the metal ion can be controlled through the chelation of phytic acid. However, our AES-R system is mainly related to the free radical mechanism because of the lack of metallic ions in the AES-R system, which is composed of only water, polysaccharides (agar), and lipids (emulsifier and soybean oil). Thus, it was assumed that phytic acid was not able to show antioxidation activity in our AES-R system.

**Figure 7:**
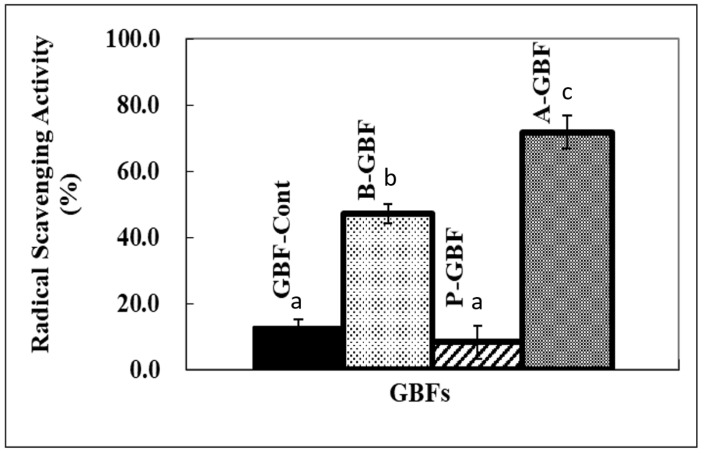
Radical scavenging activity of GBFs incorporating 1.0% antioxidants using DPPH free radical. Note that GBF-Cont represents pure GBF while A-, B-, and P- stand for ascorbic acid, BHA, and phytic acid, respectively. Bars indicate that the standard deviation and different letters (a, b, or c) are significantly different (*p* < 0.05).

In the case of ascorbic acid, the lipid oxidation was accelerated by the high quantity of ascorbic acid diffused from GBFs. This result strongly indicates that our system was fundamentally responding well to the lipid oxidation process represented by color changes of the pH indicator (resazurin). These results were relatively consistent with the radical scavenging activity test of GBFs using DPPH free radical, as shown in Figure 7. Compared to the control (pure GBF), the radical scavenging percentile of GBFs incorporating the highest levels of ascorbic acid and BHA were 71.7% and 41.7%, respectively. Herein, the changes in redness for GBFs containing ascorbic acid are not consistent with the DPPH test result. The inconsistency may be caused by the testing condition of each method. For example, the DPPH test is done with a methanol-based solvent, while the change of redness of GBFs is done in a water-based system. The different solvent system may result in different “antioxidant or prooxidant” activities. Furthermore, the radical scavenging activity is mainly related to the resonance of free radicals in antioxidant molecule structure in only one solvent system. BHA contains a conjugated ring and ascorbic acid comprises an enediol structure [25,26,27]. This implies that those two agents can become free radical scavengers due to their structures. However, all phytic acid-based GBF concentrations did not show any radical scavenging effect, as shown in the AES-R tests.

GBFs containing antioxidants can be produced by simple incorporation of active agents without severe deterioration of mechanical properties. Especially, ascorbic acid increased the gas barrier property of GBF due to its chemical structure and the oxygen scavenging effect. Phytic acid can improve mechanical and gas barrier properties of GBF due to its electrostatic cross-linking. Based on our observation, the AES-R system corresponded well to the lipid oxidation process. It could be useful to monitor the antioxidation process of food packaging systems. For further study, various antioxidation mechanisms will be tested with modification of the AES-R system. For example, adding a metal ion group may be an excellent subject to verify the effect of chelating agents such as phytic acid on the antioxidation activity of a film. The effect of the quantity of ascorbic acid may be tested because ascorbic acid plays two roles of activity, either prooxidant or antioxidant, based on the concentration. Consequently, our AES-R system using the pH indicator (resazurin) is a simple, relatively rapid, low-cost, and appropriate technology that does not require expensive instrumentation. Therefore, this simulating method will be useful for measuring the antioxidant activity of food packaging systems.

## 3. Material and Method

### 3.1. Sample and Reagent

Type B limed bone gelatin 200 bloom was a gift from Nitta Gelatin Inc. (Osaka, Japan). Resazurin sodium salt, DPPH (2,2-diphenyl-1-picrylhydrazyl), ascorbic acid, phytic acid, and BHA were purchased from Sigma Co. (St. Louis, MO, USA), Tween 80 and glycerol from Merck (Darmstadt, Germany). All chemical reagents were of analytical grade. DifcoTM plate count agar was purchased from Thermo fisher scientific (High Point, NC, USA) and adapted for making a model food system.

### 3.2. Preparation of Gelatin Biopolymer Film (GBF)

GBF solutions were prepared by dissolving 1.5 g of glycerol and 5.0 g of gelatin in 100 mL of distilled water. The concentration of each antioxidant was adjusted to 0.2, 0.6, and 1.0% of gelatin, then heated on a hot plate magnetic stirrer to 90 °C. Solutions were poured onto a 25 × 25 cm TeflonTM film coated glass plate and dried overnight at room temperature. Films were conditioned at 50% RH and 23 °C for 2 days before analysis.

### 3.3. Film Thickness

Film thickness was measured with a Digimicro MFC105 micrometer (Nikon, Japan). Measurements for testing mechanical properties were taken at five different locations on the film samples. For testing water barrier properties, measurements were taken at nine different locations. The mean thickness was used to calculate the mechanical and barrier properties of the film.

### 3.4. Measurement of Mechanical Properties

Ten specimens, 80 mm × 25 mm, were conditioned at 25 °C and 50% RH for 48 h in an environmental chamber (Hotpack Co., Philadelphia, PA, USA). An Instron Universal Testing Machine (T10000, SATEC Systems INC., Grove City, PA, USA) was used to measure the tensile strength (TS (MPa)), percentage elongation at break (E (%)), and break energy of the film (BE (in-lbf/in3)) according to a standard method (ASTM, D882-01) [53]. Initial grip separation was set at 50 mm, and crosshead speed was set at 50 mm/min. An additional 30 mm of specimen length was sufficient for testing with the Instron tensile grip without any sample slippage.

### 3.5. Measurement of Water Vapor and Oxygen Permeability

Water vapor permeability (WVP, g·cm/cm^2^s. pa) was calculated from the following Equation:

(1) $$WVP=WVTR\;\left(\frac{x}{△p}\right)$$

where WVTR is the water vapor transmission rate of films measured at 25 °C and 50% RH gradient in g/m^2^·day. x is the mean thickness of film specimens in m, and p is the actual difference in partial water vapor pressure between two sides of film specimens in Pa.

The water vapor transmission rate (WVTR) was determined gravimetrically using a modification of the ASTM, E 96-00 (2002c) [54]. According to this method, circular test cups of 4.6 cm (i.d.) × 2.1 cm deep with an exposed film area of 16.6 cm^2^ were used. Film specimens were mounted onto the open circular mouths (16.6 cm^2^) of poly(methyl methacrylate) cups filled with 16 mL of distilled water. The cups were placed in an environmental chamber set at 25 °C and 50% RH. The weight change of the cups versus time was measured and plotted. Linear regression was used to calculate the slope of a fitted straight line, which represented the WVTR. The actual RH values of the film inside the cups and the film WVP values were calculated with the correction method reported by McHugh and others [55]. The mean of the initial and final stagnant air gap height was used in the calculation.

The oxygen transmission rate was determined in an OX-TRAN 2/20 (Mocon, Inc., Minneapolis, MN, USA) at 25 °C and 50% RH conditions. Each test was done according to ASTM D3985 [43].

### 3.6. Preparation of Model Food System

The agar gel model food system was prepared similarly to the agar well diffusion method [44,56] for the bacterial test. This system contained 5% (*w/v*) agar and 30% agar of both soybean oil and Tween 80. The mix of Tween 80 (1.5 g) and soybean oil (1.5 g) was homogenized using a Vartix homogenizer at 10,000 rpm for 10 min. Agar (5 g) was added and vigorously mixed, then sterilized in the autoclave for 25 min. After taking out the agar solution, sodium azide (0.05% (*w/v*)) was added to prevent microbial spoilage. The system was neutralized up to pH 7 with diluted NaOH. Resazurin, 8 mM (10 mL), was quickly added and vigorously mixed, and then poured into plates. After 2 h of cooling in the dark, it was covered by GBFs for testing. For the calibration of our system, resazurin was individually added into only agar gel (A-R), agar and emulsifier mixed gel (AE-R), and agar, emulsifier, and soybean mixed gel (AES-R).

### 3.7. Color Measurement Corresponding to Antioxidation Activity

**Figure 8:**
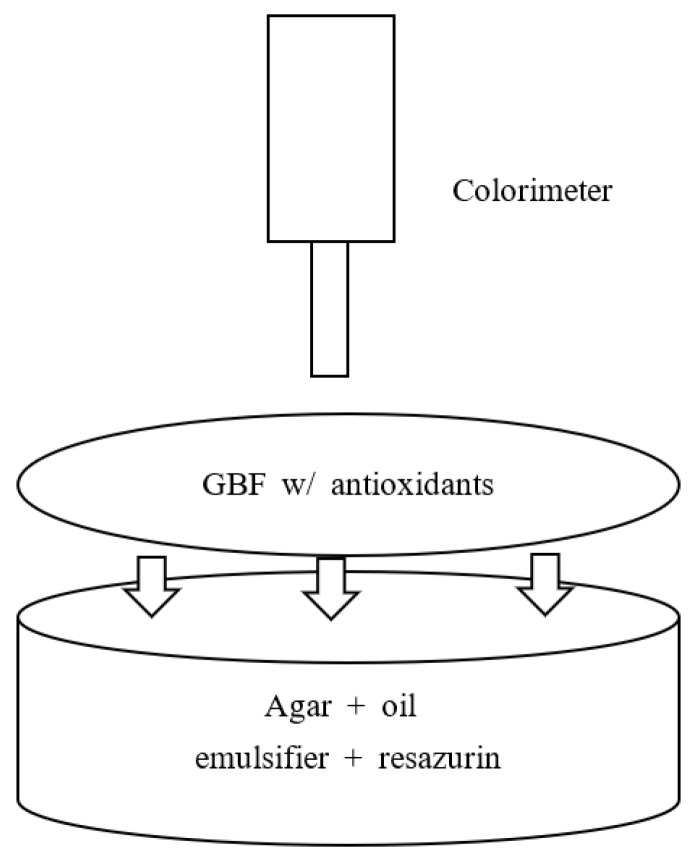
Diagram of highly lipid-based simulation food system (AES-R) for antioxidation activity test. Note that the AES-R system is composed of agar (polysaccharide), oil (soybean oil), emulsifier (Tween 80), and pH indicator (resazurin). GBF will be placed on the top surface of the AES-R system.

As shown in Figure 8, the brightness (L) and redness (a) values of both calibration systems and AES-R system covered by GBFs (AES-R w/GBFs) were measured every other day for 14 days using Minolta Colorimeter (CR-400, Minolta Corporation, Ramsey, NJ, USA). Nine points of top surface in each system were measured and calculated for the average value. Then, it was corrected by the following equation.

(2) $$corrected\;value=A_{i}-A_{0}$$

where *A_i_* is the average color value on each testing date and *A*_0_ is the initial average color value of system.

In this study, the blueness (b-value) was ignored because a systematic observation was not found, which was considered a characteristic of resazurin (blue color at neutral pH).

### 3.8. DPPH Free Radical Scavenging Test

DPPH radical scavenging activity was determined according to the method of Yen et al. [57] with a slight modification. Owing to insoluble characteristics of gelatin in a methanol-based solution, a rectangular piece of each GBF (1 cm^2^) was soaked into 5 mL of 0.05 mM DPPH in methanol. The solution was then gently shaken at room temperature in the dark for 2 h. The absorbance of the solution was measured at 517 nm using a spectrophotometer. The control was conducted in the same manner, but the sample was replaced by methanol instead. DPPH radical scavenging activity was calculated with the following equation:

(3) $$Radical\;Scavenging\;activity\;\left(\%\right)=\left(1-\left(\frac{A_{sample}}{A_{control}}\right)\right)\times 100$$

where *A_sample_* is the absorbance of the sample and *A_control_* is the absorbance of the control.

### 3.9. Statistical Analysis

Measurements were replicated three times for each type of film and model food system. Each replicated experimental unit has individually prepared films and a model food system. Statistics on a completely randomized design were performed with the analysis of variance (ANOVA) procedure in SAS (Release 6.08, SAS Institute Inc., Cary, NC, USA) software. Duncan’s Multiple Range Test (*p* < 0.05) was used to detect differences among film property and color change (L and a) mean values.

## Figures and Tables

**Table 1:** Mechanical properties (tensile, elongation at break and energy to break) of GBFs incorporating antioxidants.

Antioxidant	Conc.(%)	Tensile			Elongation			Energy to Break		
		(MPa)			(%)			(in-lbf/in^3^)		
Ascorbic acid	0.2	27.4 ^b^	±	1.3	22.8 ^b^	±	0.9	50.3 ^b^	±	3.0
	0.6	29.1 ^ab^	±	0.6	28.7 ^a^	±	1.2	62.9 ^b^	±	3.9
	1.0	26.5 ^b^	±	1.0	28.7 ^a^	±	1.8	54.7 ^b^	±	4.8
BHA	0.2	33.1 ^ab^	±	4.2	19.4 ^b^	±	3.9	59.1 ^b^	±	5.5
	0.6	29.2 ^ab^	±	3.1	21.8 ^b^	±	3.6	54.7 ^b^	±	2.0
	1.0	31.7 ^ab^	±	2.6	20.0 ^b^	±	2.1	54.6 ^b^	±	4.6
phytic acid	0.2	35.7 ^a^	±	1.0	27.5 ^a^	±	1.6	79.0 ^a^	±	5.7
	0.6	37.7 ^a^	±	1.7	24.0 ^a^	±	1.7	75.1 ^a^	±	5.2
	1.0	36.2 ^a^	±	0.7	27.6 ^a^	±	0.8	78.4 ^a^	±	3.8
Control	n/a	28.4 ^b^	±	0.5	26.2 ^a^	±	2.4	56.5 ^b^	±	9.1

Samples with different letters (^a^, ^b^) are significantly different (*p* < 0.05).

**Table 2:** Water vapor permeability of GBFs incorporating antioxidants at various concentrations (50% RH, 23 °C).

Conc.	WVP by Correction method * (ng m/m^2^ s Pa)											
	Control			Ascorbic Acid			Phytic Acid			BHA		
0.2	1.48 ^ab^	±	0.02	1.48 ^ab^	±	0.03	1.50 ^ab^	±	0.02	1.39 ^b^	±	0.05
0.4				1.40 ^b^	±	0.02	1.40 ^b^	±	0.01	1.39 ^b^	±	0.01
0.6				1.42 ^b^	±	0.05	1.29 ^c^	±	0.01	1.39 ^b^	±	0.05
0.8				1.58 ^a^	±	0.07	1.47 ^ab^	±	0.05	1.48 ^ab^	±	0.02
1.0				1.58 ^a^	±	0.04	1.59 ^a^	±	0.06	1.56 ^a^	±	

* = (39). Samples with different letters (^a^, ^b^, ^c^) are significantly different (*p* < 0.05).

## Data Availability

Not applicable.

## Supplementary Materials

The following supporting information can be downloaded at: https://www.mdpi.com/article/10.3390/molecules28052092/s1, Figure S1: The change of a value of AES-R system covered by BHA gelatin films at different concentration; Figure S2: The change of a value of AES-R system covered by ascorbic acid gelatin films at different concentration; Figure S3: The change of a value of AES-R system covered by phytic acid gelatin films at different concentration.

## Sample Availability

Samples of GBF films and AES-R systems are available from the authors.
