# Supplementary material for: A New Method to Determine Antioxidant Activities of Biofilms Using a pH Indicator (Resazurin) Model System
Source: Molecules. 2023 Feb 23;28(5):2092. doi: 10.3390/molecules28052092 (PMC10003940; doi:10.3390/molecules28052092)

Figure S-1. The change of a value of AES-R system covered by BHA gelatin films at different concentration

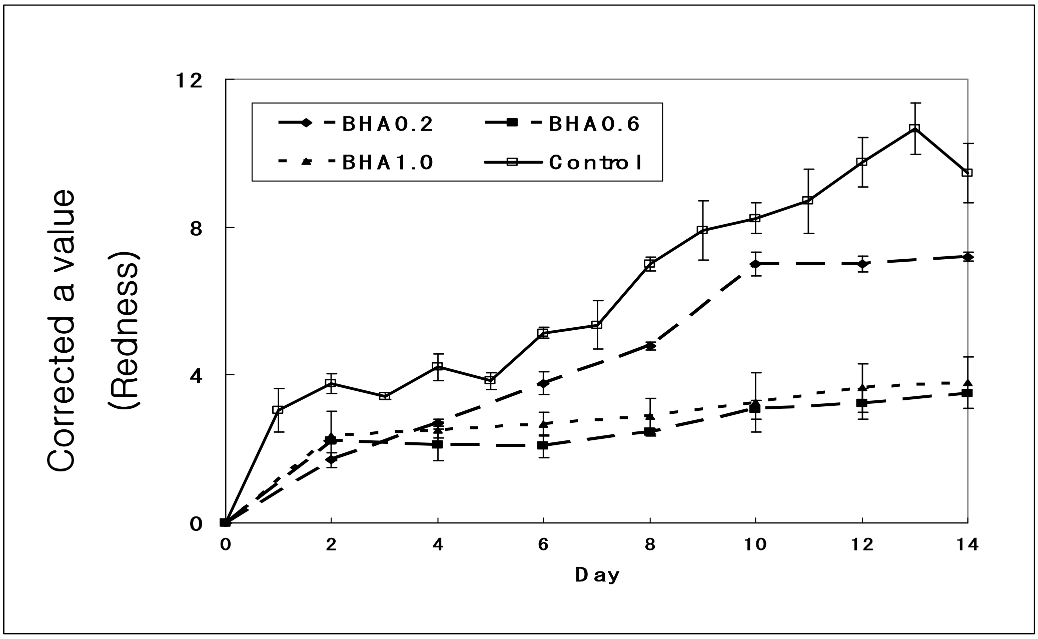

Figure S-2. The change of a value of AES-R system covered by ascorbic acid gelatin films at different concentration

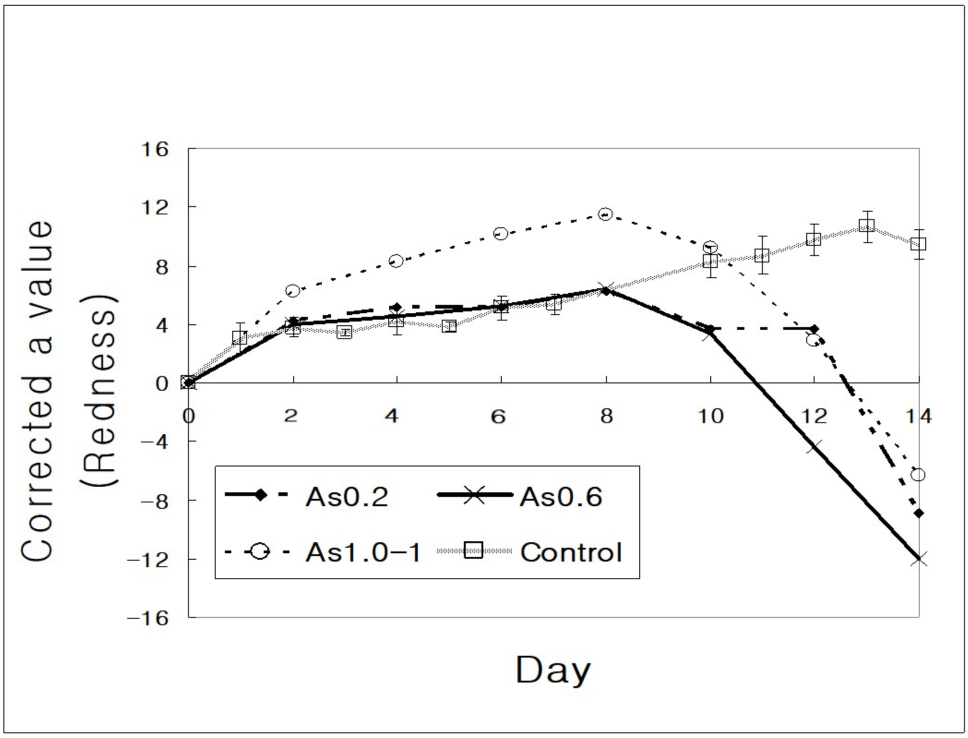

Figure S-3. The change of a value of AES-R system covered by phytic acid gelatin films at different concentration

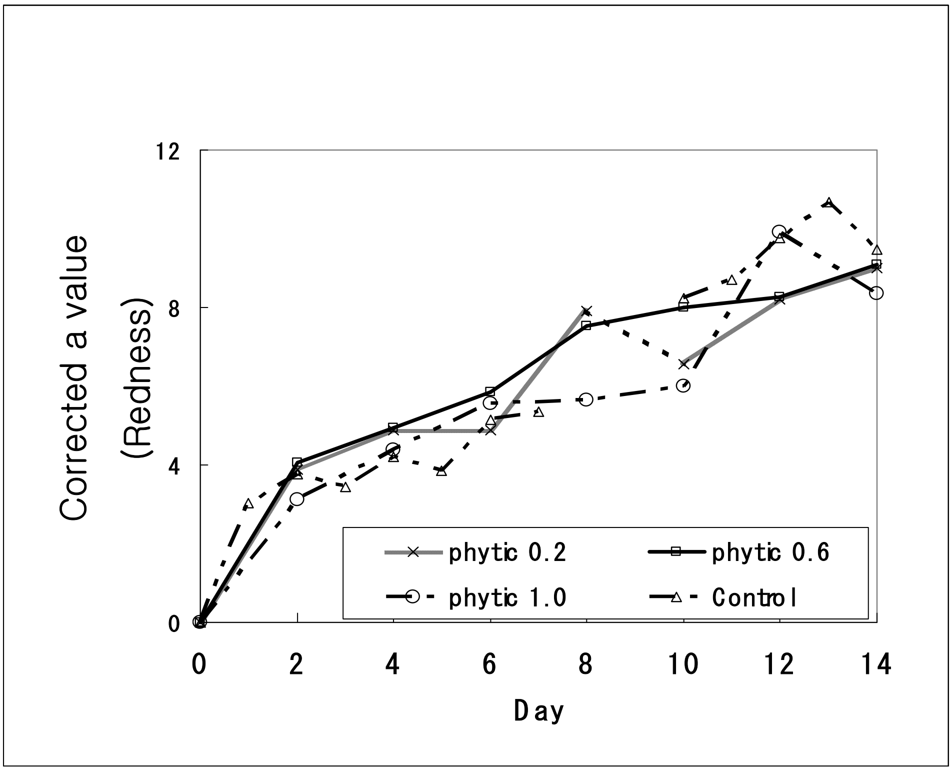

Supplement: Supplementary file 1 [file molecules-28-02092-s001.zip › molecules-2074925-supplementary.pdf]
